# How a bird is an island

**DOI:** 10.1186/1741-7007-10-53

**Published:** 2012-06-20

**Authors:** Richard Lapoint, Noah Whiteman

**Affiliations:** 1 Department of Ecology and Evolutionary Biology, University of Arizona, 1041 E Lowell St, Tucson, AZ 85721, USA

## Abstract

Replicate adaptive radiations occur when lineages repeatedly radiate and fill new but similar niches and converge phenotypically. While this is commonly seen in traditional island systems, it may also be present in host-parasite relationships, where hosts serve as islands. In a recent article in *BMC Biology*, Johnson and colleagues have produced the most extensive phylogeny of the avian lice (Ischnocera) to date, and find evidence for this pattern. This study opens the door to exploring adaptive radiations from a novel host-parasite perspective.

See research article: http://www.biomedcentral.com/1741-7007/10/52

## Adaptive radiations drive diversity

Adaptive radiations occur when lineages speciate ecologically, and are considered to be responsible for much of Earth's staggering diversity [1]. There are several classic examples of adaptive radiations: Darwin's finches in the Galapagos Islands, *Anolis *lizards in the Caribbean, Cichlids in East African lakes, and *Drosophila*, *Tetragnatha *spiders, and silverswords, all found in the Hawaiian Islands. Adaptive radiations are profoundly important to studies of evolutionary biology, and inform our understanding of how species and communities form. However, the definitions and subsequent identification of adaptive radiations have been variable [2,3]. Despite the slow progress towards a consensus definition for adaptive radiation, Johnson *et al*. [4] make a compelling case that feather lice (Phthiraptera: Ischnocera: Philopteridae) are an emerging model for the study of adaptive and convergent radiations.

We follow the conventions of several recent authors and broadly define adaptive radiations as such: all species within a radiation are descended from a common ancestor and have adapted ecologically and diversified. Species undergoing adaptive radiations experience an ecological release when new niches become available, allowing for speciation and subsequent selection for a wide range of morphologies and behaviors to adapt to their new environment. Several other factors are often invoked as being associated with adaptive radiations: a key innovation or the colonization of new adaptive space explains most of the adaptive potential of a clade; an extreme and rapid phenotypic diversification following this exploitation of a new adaptive landscape; convergence of distant lineages in similar environments; and an increase in the rate of lineage formation. Lineages arising from adaptive radiations are often found in island-like systems, including actual oceanic archipelagoes, lake systems and mountain-restricted sky islands. Alternatively, non-adaptive radiation occurs when a clade diversifies in the absence of ecological differentiation. Speciation in this case is a result of vicariance, where populations diverge into distinct species in isolation.

## Adaptive radiations of parasites

While adaptive radiations have typically been reported from classic island settings such as those mentioned above, there are other lifestyles that mimic the conditions of islands, such as parasitism. Parasitism is a remarkably successful life history strategy and has evolved numerous times across the tree of life [5]. Metazoan parasites comprise nearly 50% of all animal species and dominate food web links [6]. Despite this ecological and evolutionary dominance, there has been rampant convergent evolution in morphology, ecology and host exploitation strategies across metazoan parasites [7]. This suggests that a common set of environmental factors has selected for common phenotypes, a hallmark of adaptive evolution. For example, Clay [8] pointed out that in feather lice of birds, the 'fundamental similarity of all the Philopteridae makes it difficult to recognize whether the external characters common to two groups are due to parallel or convergent evolution or to phylogenetic relationships.' Heretofore, there have been few robust tests for adaptive radiations within parasitic clades. Parasites are often soft-bodied, and their fossil record is sparse, hampering efforts to determine the mode and tempo of evolution in parasitic species empirically. Furthermore, it has been difficult to sample enough taxa or molecular characters to robustly reconstruct the evolutionary history of an entire parasitic lineage hypothesized to be an adaptive radiation. Johnson *et al*. have presented the most inclusive and robust phylogeny of the Ischnocera to date, representing a major step forward in understanding the evolution of this radiation.

Members of the ischnoceran lice are parasitic on birds, feed mostly on feathers and dead skin, and have radiated spectacularly onto birds and mammals. The avian ischnoceran lice present many of the key features of adaptive evolution. This lineage is composed of over 2,700 species that exploit many unique niches defined by where they reside on their avian hosts; and their ecologies and phenotypes are shaped in large part by how they evade preening [9]. As Johnson *et al*. [4] suggest, avian hosts share many similarities with island systems, and the bird-feather louse system is an ideal model for studying how host-parasite interactions lead to ecologically driven speciation and could be a useful comparison for other parasite radiations.

## Repeated adaptive radiations among feather lice

**Figure 1:**
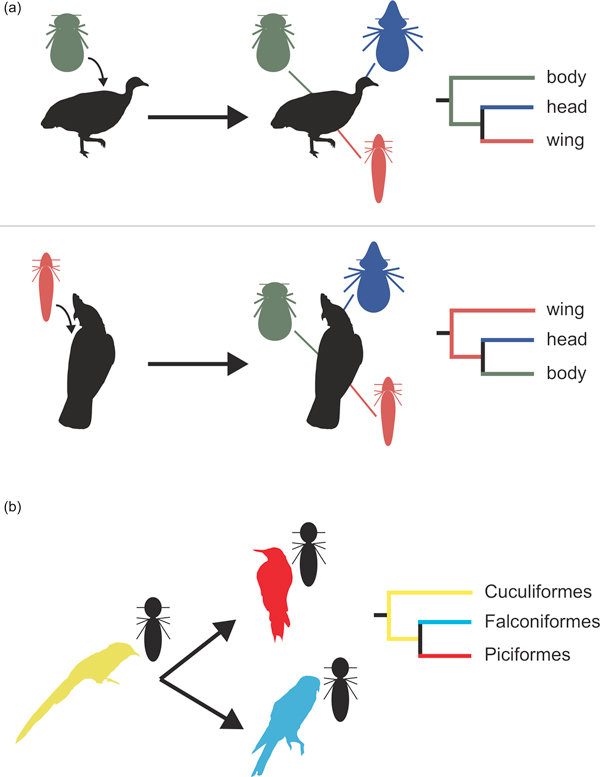
**Two types of species radiation in avian feather lice**. **(a) **Convergent adaptive radiation. After colonizing a new host, feather lice diversify to exploit the same niche via similar morphotypes. In this example from Johnson *et al*. [4], body lice on Tinamous diversify into head and wing ecotypes and wing lice on Parrots diversify into head and body lice. **(b) **Non-adaptive radiation. In this example from Johnson *et al*., a head lice lineage appears to have colonized several orders of birds (Cuculiformes, yellow; Falcinoformes, blue; Piciformes, red). This could also represent an adaptive radiation as species would still be entering a novel environment - a new host.

Johnson and coauthors have shown that avian lice display one of the more common patterns associated with adaptive radiations: morphological convergence between species adapted to similar environments, or ecotypes. This is seen in many famous examples where diverse community assemblies are made of closely related, but morphologically divergent, species filling similar or the same niches. The authors show that the feather lice represent a lineage that has radiated, repeatedly, into different host niches and many different host lineages. They provide compelling evidence that the highly divergent ecotypes based on which feathers the lice exploit - body, head, wing or generalist - have evolved over and over again as new hosts with multiple vacant niches are colonized (Figure 1a). There is also a less commonly observed pattern of vicariant speciation, where species with similar ecologies diversify in host breadth, but not in ecology (Figure 1b). This could also be a form of adaptive radiation, though, as preening behaviors differ among bird species and may force the lice to adapt in other ways. The study by Johnson *et al*. provides compelling evidence that avian lice have adaptively radiated; however, further investigation will be needed to test this.

## Future work: Going broader and deeper

To test whether a lineage displays the previously mentioned characteristics of an adaptive radiation, studies must identify how species are related and whether a change in speciation rate is due to adaptive or non-adaptive forces. Finding evidence of phenomena indicative of an adaptive radiation, such as adaptive convergence and an increased rate of morphological evolution strengthens a case for adaptive radiation. Even in the absence of an extensive fossil record, comparative phylogenetics and population-level adaptation studies are useful for testing these hypotheses. Phylogenetic methods can test lineages for patterns consistent with adaptive radiations. However, they are less effective when sampling is incomplete and results are complicated by the difficulty in inferring a supported and robust phylogeny in recent and rapid radiations. Adaptation experiments strengthen phylogenetic results by testing how these patterns arose in contemporaneous species. By combining these two approaches future research will further illuminate the nature of this radiation.

While the study by Johnson *et al*. is by far the most rigorous phylogenetic treatment of the Ischnocera to date, denser taxon sampling is required to rigorously test whether replicate ecological radiations or vicariant diversification across hosts is more prevalent among avian lice. If sampling is increased in even a few feather lice lineages with multiple phenotypes or hosts, the pattern of speciation will be more evident. While the authors conclusively show that head, body and wing ecotypes have repeatedly evolved independently, quantifying how over- or under-dispersed divergent lineages are would identify how important replicate radiation was to avian lice diversification. Another avenue of research would be to test if certain ecotypes increase the chance of dispersal to new hosts, or constrain the ability to switch to other hosts. By more exhaustively sampling taxa from multiple host lineages it should be possible to explore whether a complete community assembles from a common ancestor every time a new host is colonized, and how long this process takes. This additional information will be important for evaluating and comparing the mode of diversification in the ischnoceran lice in light of other radiations.

It may also be possible to perform microevolutionary experiments within this group. Identifying what variation in head or body shape of the lice is present within populations and whether there is Mendelian or quantitative genetic variation underlying it would be useful. This information would aid the design of parasite transfer experiments to test if ecotypes will re-evolve in the presence of empty niches. For simplicity's sake, this would be especially informative if identified in young radiations endemic to a single host family, where shared variance may be due to identity by descent. The feather lice found on the Tinamous are exceedingly diverse and may present just such a system for future work [10]. Clay [8] noted (p. 143) 'The genera of the *Heptapsogaster*-complex found on the Tinamidae are believed to be descended from a single ancestral stock on this group which has branched out to fill the different ecological niches on the body of the bird; the species now have a superficial resemblance to the unrelated occupants of similar niches on other orders.'

## Lice as a model system for adaptive radiations

The ischnoceran feather lice have successfully radiated onto many different host lineages and into several different phenotypes to exploit the available niches. Johnson *et al*. show that this diversification is largely due to replicate adaptive radiations, resulting in convergent ecologies and phenotypes, a common hallmark of other classic adaptive radiations. This study is the foundation for the development of ischnoceran lice into a model system for host-parasite adaptive radiations, an area of research that has been relatively unexplored, but may underlie much of the diversity of life.
